# Awareness and Use of HIV Self-Testing Among Men Who Have Sex With Men Remains Low in Spain 2 Years After Its Authorization

**DOI:** 10.3389/fpubh.2022.888059

**Published:** 2022-06-17

**Authors:** Juan-Miguel Guerras, Juan Hoyos, Luis de la Fuente, Marta Donat, José Pulido, Luis Sordo, Patricia García de Olalla, María-José Belza

**Affiliations:** ^1^Centro Nacional de Epidemiología, Instituto de Salud Carlos III, Madrid, Spain; ^2^CIBER Epidemiologia y Salud Pública, Madrid, Spain; ^3^Independent Researcher, Madrid, Spain; ^4^Departamento de epidemiología y bioestadística, Escuela Nacional de Sanidad, Instituto de Salud Carlos III, Madrid, Spain; ^5^Departamento de Salud Pública y Materno-Infantil, Universidad Complutense de Madrid, Madrid, Spain; ^6^Servicio de Epidemiología, Agència de Salut Pública de Barcelona, Barcelona, Spain

**Keywords:** early diagnosis, HIV self-testing (HIVST), self-diagnosis, infectious disease, men who have sex with men (MSM)

## Abstract

**Objectives:**

HIV self-testing has been available in Spain since 2017 as a diagnostic tool to promote earlier diagnosis. We aimed to assess awareness and previous use of HIV self-testing in a sample of men who have sex with men (MSM) recruited online more than 2 years after its legal authorization in Spain.

**Methods:**

We analyzed 5,492 MSM recruited between May and July 2020 in gay dating apps/websites and other social networks. We estimated the proportion of participants who were aware of the existence of HIV self-testing and the proportion who reported previous use. To identify factors associated with both outcomes we built two Poisson regression models. Among those who reported previous use we described several aspects related to their last self-test.

**Results:**

Awareness of HIV self-test was reported by 29.7% and its previous use by 5% of participants. Awareness was independently associated with recruitment in gay dating apps/websites, being ≥40 years old, born in Spain-other European countries, having higher educational level, living in medium-small municipalities and living sex life openly. Independent associations were also found with having received a sexually transmitted infection diagnosis (STI) or an HIV negative test in the last 12 months, and being HIV positive. Use was significantly higher among participants who were paid for sex or diagnosed with an STI in the last 12 months and who received their last HIV test in the preceding year. Self-testing occurred recently, with kits acquired at pharmacies and carried out alone.

**Conclusion:**

Awareness and use probably have not increased sufficiently in order to make a relevant impact to the promotion of early HIV diagnosis. Additional efforts, mainly focused on less favored MSM, should be made to take better advantage of the possibilities offered by this testing option.

## Introduction

In 2020, 1.925 new HIV cases were reported to the Spanish surveillance system of which 52.2% were acquired through sex between men (1). Early diagnosis is the gateway to treatment which reduces morbidity (2, 3) and mortality (4) and drastically reduces the probability of transmission to an uninfected individual (5). Early diagnosis is in fact one of the main elements of the UNAIDS 95-95-95 strategy to end the HIV epidemic. According to this strategy, by 2030, 95% of people living with HIV should be diagnosed, 95% of whom should be on treatment, 95% of whom should have undetectable viral load (6).

Testing in Spain is offered confidentially and free of cost at all levels of the national health system. It is also offered in sexual health clinics which are public, free, low threshold facilities oriented toward the most vulnerable population groups (7). Outside clinical settings, rapid testing is also offered free of cost in Community Based Organizations (8) and in community pharmacies (9). However, in Spain 16.5% of MSM living with HIV remain undiagnosed (10) and delayed diagnosis is still common with 39.6% of newly diagnosed MSM in 2020 presenting at a late stage of infection (CD4 count of <350 mm3) (1).

HIV self-testing is the latest incorporation to already existing testing options and aims at making HIV diagnosis more accessible. In Spain, it was authorized in December 2017 (11) and can now be purchased at pharmacies at a cost of 25–30 euros. In this methodology, individuals need to perform their own test and interpret the results. Self-testing kits can be blood or saliva based and, if reactive, confirmatory testing is required. Clinical trials have proven its capacity to increase testing frequency among MSM (12–14) and online studies conclude that self-testing is highly acceptable to MSM (15) who are largely in favor of it (16).

Nevertheless, its real use is difficult to ascertain since no data is available on the number of self-testing kits sold. Additionally, surveillance data does not publish information on the type of test used in newly diagnosed individuals. Self-reported data on awareness about the existence of self-testing and previous use is one way of overcoming this data shortage. In Spain, a number of studies assessed awareness about the existence and use of self-testing by MSM in online recruited samples (15, 17) and among attendees of a rapid testing program (18). All of them found lower levels of awareness than in other European countries (17, 19) whereas the level of use also sat in the lower range (17, 19). All the aforementioned studies present data collected before self-testing authorization in the studied countries. Media attention, promotion campaigns and mouth to ear following authorization probably led to higher levels of awareness but very few studies have assessed this aspect post-authorization. The only study that evaluated both outcomes after the legal authorization of HIV self-testing was conducted in Spain and is in line with this hypothesis: level of awareness and use was substantially higher than in previous studies conducted in Spain (20). However, this study analyzed a sample of MSM who were seeking to be tested for HIV in Madrid and Barcelona, the two largest cities of Spain. Thus, by definition, it did not include participants who had never sought testing and those who already were HIV positive. Additionally, the sample was comprised almost exclusively by participants who lived in the aforementioned major cities both of which are more than 1.000.000 inhabitants and did not include MSM from medium-sized cities and rural areas who might have different testing needs.

In this study, we analyzed an online sample of MSM recruited through gay dating websites/apps and other social networks more than 2 years after the authorization of HIV self-testing in Spain and assessed their awareness of its existence as well as the level of previous use and describe several aspects related to the self-testing process.

## Methods

### Participants

We performed an online based cross-sectional study between May and July 2020. We included 5,492 participants who were male at birth, who reported ever having had anal sex with another man and who were legally old enough to have sex at the moment of participation.

No sample size calculation was performed. We used non-probabilistic convenience sampling to recruit the largest possible sample of MSM.

### Recruitment and Data Collection Instrument

Recruitment was mainly carried out using two dissemination methods. First, participants were invited to participate through personal messages and promotional banners distributed through gay dating apps and websites. Invitations were only presented to participants accessing from Spain. Secondly, we also contacted several Spanish youtubers and Instagram influencers with LGTB oriented content who agreed to promote the study (hereafter Social networks other than dating apps). We provided unique links to each recruitment site to identify the recruitment site of each participant. Those interested in participating were directed to an initial screen where they were informed about the aim of the study, the funding source and the approximate length of the questionnaire (10–15 min). To avoid multiple participation, the initial screen also included a request to only complete the questionnaire once if invitation was received through various ways. Additionally, to further limit the possibility of multiple responses from one individual, we used the option given by the software of only allowing the completion of one questionnaire per electronic device. After reading the initial screen, those who decided to participate were redirected to the questionnaire after checking a box to confirm that they were old enough to legally have sex in Spain (16 years old) and that they wanted to participate in the study. No incentive was offered in exchange for participation which also limited the chances of multiple participation.

Data was collected through a self-administered questionnaire that included questions assessing sociodemography, outness, sex of lifetime sexual partners, sexual behavior in the last 12 months, history of Sexually Transmitted Infections (STI), testing history and HIV serostatus. It also included a section to assess whether participants knew about the existence of self-testing (awareness) and if they had already used it at the moment of survey completion (use). We also included questions to asses overall sexualized drug use and “Chemsex.” The latter was defined as having taken mephedrone and/or methamphetamine and/or gamma hydroxybutyrate (GHB) (21) within 6 h before or during anal sex. To assess sex of lifetime sexual partner, participants were asked to choose one of the following options: (a) I have never had sex neither with men nor with women, (b) I have had sex only with women, (c) I have had sex more often with women, but at least once with a man, (d) I have had sex equally with men and women, (e) I have had sex more often with men, but at least once with a woman and (f) I have had sex only with men. Those who chose answer (a) or (b) were excluded from participation.

To assess economic status, we asked participants to choose one of the following response options: (a) “Very comfortable,” (b) “Comfortable,” (c) “It's tight, I have to be careful to make ends meet,” (d) “I make ends meet with difficulty” (e) “I make ends meet with debts.” Based on this question we built a three category variable: options (a) and (b) were collapsded into the “Comfortable-very comfortable” category, (d) and (e) into the “difficult” category and option (c) conformed the “tight” category.

The questionnaire was designed in the context of a research Project (the Methysos Project) which focused on MSM and had HIV-self testing among its several areas of interest. The research group, developed a draft version of the questionnaire which was shared with several experts on the field who critically reviewed it. The suggested changes were discussed and incorporated into the questionnaire. Afterwards, the questionnaire was piloted to check its internal consistency.

Awareness and use comprised our main outcomes. Awareness was assessed with the following question: “Did you know that in Spain you can buy an HIV self-test at the pharmacy or parapharmacy without the need of a prescription?” Participants had to choose between the following response options: (1) No, I did not now, (2) I had heard something but was not sure and (3) Yes, I knew. Use was assessed by asking: “Have you ever tested for HIV using a self-testing kit?” In this case, participants had to choose between Yes and No. For participants who reported previous use, we included a set of questions to assess the number of self-tests performed in the past, time since last use, place of acquisition of the last self-testing kit and the person with whom participants were with during their last self-testing episode. We created a three category variable to assess serostatus and testing history. The first category is comprised by participants who did not meet current recommendations for MSM of undergoing HIV testing at least every 12 months (22). Never-testers and participants who reported having tested more than 12 months ago were included in this category. The second category was comprised by participants who received their last test in the previous 12 months. The third and last category was comprised by those who reported being HIV positive. The survey was anonymous and confidential and no variables enabling personal identification were collected. The study was approved by the Research Ethics Committee of the Instituto de Salud Carlos III (CEI PI 35_2020-v3).

### Data Analysis

We first performed a descriptive analysis of the main characteristics of the sample. Secondly, we estimated the proportion of participants who reported awareness and use of HIV-self testing by relevant independent variables. Awareness of self-testing was collapsed in a two-category variable: (1) “Yes, I knew”; (2) “No, I did not” or “I had heard something but was not sure.” To estimate the factors associated with awareness and with use, we used Poisson regression with robust variance in the framework of generalized linear models' analysis (23, 24) to calculate the crude and adjusted prevalence ratios (PRs) with their 95% confidence intervals (95% CI). We initially included all the relevant variables with a significance level ≤ 0.20 and employed the minimum Akaike information criterion and the minimum Bayesian Schwartz information criterion for model comparisons and to select the optimal model. For those who reported having used it in the past, we assessed the number of times used, time since last use, where was the last self-test acquired and who was he with the last time they self-tested.

## Results

### Main Characteristics of Participants

Seventy-two percent of the study participants were recruited through gay dating apps and websites, and 26.6% through Social networks other than dating apps. Forty-five percent were ≥40 years of age, 82.8% were born in Spain and 54.7% had finished a university degree at the moment of participation. Approximately 65% reported having a comfortable or very comfortable economic situation and 31.6% lived in cities of >1.000.000 inhabitants. More than half (54.4%) lived their sex-life with other men openly. Regarding sexual behaviors, 63.2% reported only having had sex with other men in the past (and never with women), 71.8% were involved in condomless anal sex in the last 12 months and 23.9% and 22.5% had paid and received money in exchange for sex in the same time period. Almost half (46.2%) reported having been diagnosed with an STI in the past (16.2% in the last 12 months) and 10% had been involved in Chemsex in the previous 12 months. An HIV positive serostatus was reported by 14% whereas 55.2% received their last negative test in the last 12 months and 30.8% had never been tested or had received their last negative test >12 months ago (of whom 15.3% were never testers and 15.5% had tested more than 12 months ago) (Table 1).

**Table 1:** Main characteristics of study participants (*N* = 5,492).

	***N***	**%**
**Recruitment site**		
Gay dating apps/websites	3,954	72.0
Social networks other than dating apps	1,460	26.6
Other^*^	78	1.4
**Age at the moment of participation (years)**		
<30	1,534	27.9
30–39	1,489	27.1
≥40	2,469	45.0
**Place of birth**		
Spain	4,547	82.8
Latin America	736	13.4
European countries other than Spain	164	3.0
Other countries	45	0.8
**University education**	3,003	54.7
**Economic situation**		
Comfortable-very comfortable	3,547	64.7
Tight	1,488	27.1
Difficult	448	8.2
**Inhabitants of place of residency**		
More than 1,000,000 inhabitants	1,731	31.6
100,000–1,000,000	1,812	33.1
10,000–99,999	1,283	23.4
<10,000	646	11.8
**Lives sex life with other men...**		
Openly	2,984	54.4
Discreetly	2,009	36.6
Hidden/In total secrecy	497	9.1
**Has had sex only with men**	3,472	63.2
**Number of male sexual partners with whom he had condomless**		
**anal sex (last 12 months)**		
None	1,546	28.2
One	1,550	28.2
2–4	1,228	22.4
5–9	417	7.6
10 or more	749	13.6
**Has paid for sex in the last 12 months**	1,307	23.9
**Has been paid for sex in the last 12 months**	1,234	22.5
History of STI^**^		
Never diagnosed with an STI	2,924	53.8
Diagnosed in the last 12 months	878	16.2
Diagnosed more than 12 months ago	1,634	30.1
Has been involved in chemsex (last 12 months)^***^	520	10.0
**HIV testing history/serostatus**		
Never tested & last tested >12 months ago	1,690	30.8
Last tested <12 months ago	3,029	55.2
HIV positive	768	14.0

*% estimated over cases with valid data*.

* *Public health agency and NGOs*.

** *STI, Sexually transmitted infections*.

*** *Mephedrone and/or methamphetamine and/or gamma hydroxybutyrate (GHB) within 6 h before or during anal sex*.

### Awareness of the Availability of HIV Self-Testing Kits in Pharmacies Without the Need of a Prescription

Overall, 29.7% of all MSM reported being aware of the existence of HIV self-testing (Table 2). On the multivariable analysis, awareness was associated with recruitment in gay dating websites/apps [Adj.PR = 1.2; (CI 95%: 1.1–1.3)] and being ≥ 40 years old [Adj.PR = 1.2; (CI 95%: 1.0–1.3)]. Compared to participants born in Latin America, awareness was higher among those born in Spain [Adj.PR = 1.5; (CI 95%: 1.3–1.7)] and other European countries [Adj.PR = 1.4; (CI 95%: 1.1–1.8)]. Compared to those who reported having up to compulsory secondary education, awareness was higher among those who had upper secondary [Adj.PR = 1.2; (CI 95%: 1.0–1.4)] or university education [Adj.PR = 1.3; (CI 95%: 1.1–1.6)]. Living in cities of under 1.000.000 inhabitants [100.000–1.000.000 Adj.PR = 1.2; (CI 95%: 1.1–1.4); 10.000–99.999 Adj.PR = 1.2; (CI 95%: 1.1–1.3); <10.000 Adj.PR = 1.2; (CI 95%: 1.0–1.3)] and living sex life with other men openly [Adj.PR = 1.4; (CI 95%: 1.1–1.7)] were also associated with higher awareness. Finally, it was higher among those reported having received an STI diagnosis in the last 12 months [Adj.PR = 1.2; (CI 95%: 1.1–1.4)] and among those who reported having received their last HIV negative test <12 months ago [Adj.PR = 1.5; (CI 95%: 1.4–1.7)] or being HIV positive [Adj.PR = 1.9; (CI 95%: 1.6–2.2)].

**Table 2:** Proportion of participants who were aware of the existence of HIV self-testing and associated factors (*N* = 5,492).

	***N***	**%**	**cPR**	**95% CI**	**aPR**	**95% CI**
Total	1,632	29.7				
**Recruitment site**						
Social networks other than dating apps and others^*^	359	23.3	1.0		1.0	
Gay dating apps/websites	1,273	32.2	1.4	1.2–1.6	1.2	1.1–1.3
**Age at the moment of participation (years)**						
<30	372	24.3	1.0		1.0	
30–39	441	29.6	1.2	1.1–1.4	1.1	0.9–1.2
≥40	819	33.2	1.4	1.2–1.5	1.2	1.0–1.3
**Place of birth**						
Latin America	156	21.2	1.0		1.0	
Spain	1,413	31.1	1.5	1.2–1.7	1.5	1.3–1.7
Other European countries	52	31.7	1.5	1.1–2.0	1.4	1.1–1.8
Other countries	11	24.4	1.2	0.6–2.1	1.2	0.7–1.9
**Education**						
Up to compulsory secondary education	131	24.5	1.0		1.0	
Upper secondary education	541	27.7	1.1	0.9–1.4	1.2	1.0–1.4
University education	959	31.9	1.3	1.1–1.6	1.3	1.1–1.6
**Economic situation**						
Difficult	113	25.2	1.0			
Tight	401	26.9	1.1	0.9–1.3		
Comfortable-very comfortable	1,118	31.5	1.2	1.0–1.5		
**Inhabitants of place of residency**						
More than 1,000,000 inhabitants	476	27.5	1.0		1.0	
100,000–1,000,000	587	32.4	1.2	1.0–1.3	1.2	1.1–1.4
10,000–99,999	381	29.7	1.1	0.9–1.2	1.2	1.1–1.3
<10,000	183	28.3	1.0	0.9–1.2	1.2	1.0–1.3
**Lives sex life with other men...**						
Hidden/In total secrecy	109	21.9	1.0		1.0	
Discreetly	533	26.5	1.2	1.0–1.5	1.1	0.9–1.3
Openly	988	33.1	1.5	1.2–1.8	1.4	1.1–1.7
**Number of male sexual partners with whom he had condomless anal sex (last 12 months)**						
None	463	29.9	1.0		1.0	
One	386	24.9	0.8	0.7–1.0	0.8	0.8–1.0
2–4	362	29.5	1.0	0.9–1.1	0.9	0.8–1.0
5–9	132	31.7	1.1	0.9–1.3	0.9	0.8–1.1
10 or more	287	38.3	1.3	1.1–1.5	0.9	0.8–1.1
**Has been paid for sex in the last 12 months**						
No	1,227	28.9	1.0			
Yes	401	32.5	1.1	1.0–1.3		
History of STI^**^						
Never diagnosed with an STI	735	25.1	1.0		1.0	
Diagnosed more than 12 months ago	537	32.9	1.3	1.2–1.5	1.1	1.0–1.2
Diagnosed in the last 12 months	343	39.1	1.6	1.4–1.8	1.2	1.1–1.4
Has been involved in chemsex^***^(last 12 months)						
No	1,364	29.2	1.0			
Yes	187	36.0	1.2	1.1–1.4		
**HIV testing history/serostatus**						
Never tested & last tested >12 months ago	326	19.3	1.0		1.0	
Last tested <12 months ago	974	32.2	1.7	1.5–1.9	1.5	1.4–1.7
HIV positive	328	42.7	2.2	1.9–2.6	1.9	1.6–2.2

*% estimated over cases with valid data. cPR, Crude Prevalence Ratio; aPR, Adjusted Prevalence Ratio; CI, Confidence Interval*.

* *Others includes, Public health agency and NGOs*.

** *STI, Sexually transmitted infections*.

*** *Mephedrone and/or methamphetamine and/or gamma hydroxybutyrate (GHB) within 6 h before or during anal sex*.

### Previous Use of HIV Self-Testing Kits

Five percent reported having already used an HIV self-test before the day of survey completion. On the multivariable analysis, previous use was higher among participants who reported having been paid for sex in the last 12 months [Adj.PR = 1.4; (CI 95%: 1.1–1.8)], in those who reported having been diagnosed with an STI in the last 12 months [Adj.PR = 1.4; (CI 95%: 1.0–1.8)] and among those who reported having been tested for HIV in the previous 12 months [Adj.PR = 4.7 (CI 95%: 3.1–7.1)] (Table 3). Among participants who reported previous use of a self-test, over half (52.2%) reported having used it only once, 65.9% used their last self-test in the previous 12 months, 62.4% acquired it over the counter at a pharmacy and 71.6% used it alone (Table 4).

**Table 3:** Proportion of participants who had used a self-testing kit in the past and associated factors (*N* = 5,488).

	***N***	**%**	**cPR**	**95%CI**	**aPR**	**95%CI**
Total	275	5.0				
**Recruitmen site**						
Social networks other than dating apps and others^*^	66	4.3	1.0			
Gay dating apps/websites	209	5.3	1.2	0.9–1.6		
**Age at the moment of participation (years)**						
<30	80	5.2	1.2	0.9–1.6		
30–39	89	6.0	1.4	1.1–1.8		
≥40	106	4.3	1.0			
**Place of birth**						
Latin America	31	4.2	1.0			
Spain	226	5.0	1.2	0.8–1.7		
European countries other than Spain	14	8.5	2.0	1.1–3.8		
Other countries	4	8.9	2.1	0.7–6.0		
**Education**						
Up to compulsory secondary education	17	3.2	1.0			
Upper secondary education	88	4.5	1.4	0.8–2.4		
University education	169	5.6	1.8	1.1–2.9		
**Lives sex life with other men...**						
Hidden/In total secrecy	14	2.8	1.0			
Discreetly	85	4.2	1.5	0.9–2.6		
Openly	175	5.9	2.1	1.2–3.6		
**Number of male sexual partners with whom he had condomless anal sex (last 12 months)**						
None	66	4.3	1.0			
One	63	4.1	1.0	0.7–1.3		
2–4	58	4.7	1.1	0.8–1.6		
5–9	29	7.0	1.6	1.1–2.5		
10 or more	59	7.9	1.8	1.3–2.6		
**Has paid for sex in the last 12 months**						
No	198	4.8	1.0			
Yes	77	5.9	1.2	1.0–1.6		
**Has been paid for sex in the last 12 months**						
No	193	4.5	1.0			
Yes	80	6.5	1.4	1.1–1.9	1.4	1.1–1.8
**Unprotected intercourse with women (last 12 months)**						
No	255	4.9	1.0			
Yes	20	6.6	1.3	0.8–2.1		
History of STI^**^						
Never diagnosed with an STI	129	4.4	1.0		1.0	
Diagnosed more than 12 months ago	74	4.5	1.0	0.8–1.4	1.0	0.7–1.3
Diagnosed in the last 12 months	68	7.8	1.8	1.3–2.4	1.4	1.0–1.8
**HIV testing history/serostatus**						
Never tested & last tested >12 months ago	25	1.5	1.0		1.0	
Last tested <12 months ago	230	7.6	5.1	3.4–7.8	4.7	3.1–7.1
HIV positive	20	2.6	1.8	1.0–3.2	1.4	0.7–2.6

*% estimated over cases with valid data. cPR, Crude Prevalence Ratio; aPR, Adjusted Prevalence Ratio (adjusted by age); CI, Confidence Interval*.

* *Others includes, Public health agency and NGOs*.

** *STI, Sexually transmitted infections*.

**Table 4:** Number of self tets performed, times since last self-testing episode, place of acquisition and company during last episode among participants who reported having used a self-test in the past (*N* = 275).

	***N***	**%**
Number of self-testing kits used^*^		
One	143	52.2
Two	51	18.6
Three or more	80	29.2
Time since last self-test^*^		
≤ 12 months	180	65.9
>12–24	49	17.9
>24 months to 5 years	34	12.5
>5 years ago	10	3.7
Where was the last self-test acquired^*^		
At a pharmacy (over the counter)	171	62.4
At an online pharmacy	36	13.1
Vending machine	0	0.0
At a country (other than Spain) where it is legally sold	19	6.9
Purchased it on the internet without having certainty about its legality	7	2.6
Others	41	15.0
**With whom did he self-test last time**		
Alone	197	71.6
Stable partner	25	9.1
Occasional partner	8	2.9
Friend	27	9.8
Family member	5	1.8
CBO/NGO staff	6	2.2
Health profesional	3	1.1
Others^**^	4	1.5

*% estimated over cases with valid data*.

* *Due to missing values, the total does not add up to 275*.

** *Others include: pharmacist, psychologist and “I do not remember”*.

## Discussion

Awareness and previous use in this online sample of MSM recruited more than 2 years after its legal authorization in Spain remains low. Awareness was found to be higher in participants recruited in gay dating websites/apps, older and European individuals, with educational levels above compulsory secondary education, who lived in medium to small sized municipalities and who lived their sex life with other men in an open manner. Awareness was also higher among those who reported having been diagnosed with an STI in the last 12 months and among HIV positive individuals. Use was higher in those who reported having been paid for sex or diagnosed with an STI in the last 12 months and among participants who received their last negative HIV test in the last 12 months.

We recruited a large and varied online sample of MSM using gay dating apps-websites and other social networks. Thus, we were able to include relevant subgroups such as MSM living in medium and small sized municipalities as well as never tested and HIV positive individuals. The overall awareness rate was notably higher than in previous studies conducted before its legal authorization in Spain (15, 17) and other European countries such as Denmark, Greece, Portugal, Romania or Slovenia and very similar to that found in Germany, Belgium (17) and France (19). The higher level of awareness found in our study—when compared to pre-approval studies conducted in Spain—is probably explained by the media attention and promotion that followed its approval. In fact, in the only study conducted post-approval, awareness among attendees of sexual health clinics and community-based testing programs in Madrid and Barcelona (20) was very similar to what has been found in the present study. However, in spite of its growth, awareness is still very low and additional efforts need to be made to improve it in MSM. In this sense, the popularization of COVID 19 self-testing devices could have increased the readiness of both the general and MSM populations for self-diagnostic technologies and offers an opportunity to roll-out effective self-testing promotion campaigns.

Awareness is relevant because it is a necessary condition to access self-testing. Our multivariable analysis revealed that it is higher in participants recruited through gay dating websites suggesting the need of promoting this methodology in other online settings serving MSM with a different profile such as YouTube, Instagram and other social networks. Concerning sociodemography, increased awareness was found among participants who were over 40 years of age, born in European countries and with higher educational level. These findings highlight the need of raising awareness among less favored groups such as Latin-American migrants and those with lower educational level to reduce testing access inequities. Although the variable assessing economic situation was not retained in the final model, when considering the promotion of self-testing among less favored subpopulations it is important to note, that regardless of the level of awareness, the current price of 25–30 euros could be an important barrier as has been suggested in previous studies (25).

The higher level of awareness found in residents of medium and small size cities and towns is an encouraging finding since MSM from less populated municipalities could have lower access to HIV testing than MSM residing in larger cities. Home-based testing has been highlighted as a preferential testing option by rural MSM in studies (26–28) conducted in the US. This finding needs to be confirmed in the European context but the higher levels of awareness found in our study could be an expression of the need of increased access to HIV testing in this subgroup of MSM. Awareness was also higher among MSM who lived their sex life with other men in an open manner which is in line with previous studies (20) and highlights the need of increasing it among less open MSM. In fact, the latter could be facing stigma and discrimination related barriers and the privacy and anonymity offered by self-testing could facilitate access to testing (29).

The higher awareness found among those diagnosed with an STI in the last 12 months could reflect a “worried well” situation since higher rates of STI are generally associated with increased risk of condomless sex. However, in our sample, we did not find associations between higher rates of condomless anal sex and awareness.

The association was especially intense among HIV positive MSM. This is the first time that awareness is assessed in already diagnosed individuals who comprise a non-negligible subgroup of the MSM population. Although, they cannot directly benefit from this testing methodology HIV positive individuals could recommend it to peers, friends and sexual partners and could help to increase awareness among MSM. On the other hand, it is important to underline the low levels of awareness found among undertested MSM. This is a relevant finding as data from modeling studies highlight the importance of specifically targeting never testers as the most efficient way of averting new infections through HIV self-testing strategies (30).

Similar to awareness, the proportion of participants who reported previous use was higher than in online studies conducted among MSM before its authorization in Spain (15, 17) and other European countries (17) and very similar to a study conducted in Spain after its approval (20). Nevertheless, previous use still remains very low. The increase of use of HIV self-testing could lead to a rise of testing frequency in MSM and, if accompanied of prompt linkage to care, could contribute to curbing the epidemic in this population group. There are several possible strategies to increase the low use found in our study. First, use will not increase before MSM are made aware of the existence of this testing option and, as noted before, awareness in Spain is still low. If promotion of HIV self-testing is left to mouth to ear, use will probably increase very slowly. Promotion campaigns focusing on those MSM groups who do not know about the existence of self-testing, should be conducted to raise the number of MSM who use a self-testing kit to check their serostatus. In this sense, the new Spanish plan for the prevention and control of HIV and STIs (2021–2030) includes the visibilization of self-testing (31) as one of its activities. Second, to overcome the aforementioned price barrier, partly or fully publicly funded distribution of HIV self-testing could increase its use (14) especially in less favored individuals and in MSM with ongoing sexual behaviors for whom a test each 3 months is recommended. Third, diversification of distribution strategies could also increase self-testing. In our sample, most MSM acquired their last self-test over the counter at a pharmacy but online marketing and distribution could help improve access to self-testing as suggested by a number of studies (12). This is especially important for MSM living in rural areas who can have limited access to pharmacy offices. Satisfaction with the self-testing experience will not affect overall use but will influence the decision on whether to use a self-test again. In our study we did not assess user satisfaction but almost half of the participants who had used a self-test reported having used it more than one time.

The use of self-testing was higher in certain subgroups. Thus, we found a higher use in two high risk groups such as those who had been paid for sex and among MSM who received an STI diagnosis in the last 12 months. For sex workers, HIV self-testing could offer the possibility of checking their (and their clients) serostatus before engaging in sexual encounters. However, this practice is risky due to longer window periods and reduced accuracy of self-testing during acute infection (32) that can lead to false-negative results in a highly infectious stage (33). Similar to knowledge, use was substantially higher among those who received their self-test in the last 12 months suggesting the need of promoting self-testing especially among undertested individuals as a way of increasing the fulfillment of testing recommendations.

Results are not without limitations. Although we made an effort to recruit MSM from a varied range of sites, this is a convenience sample and extrapolating conclusions from this study to other MSM populations should be made with extreme caution. We did not ask participants about their sexual orientation since our approach to sexuality was strictly behavioral. We could not ascertain if any of the HIV positive participants were diagnosed as a consequence of using a self-test since the site where diagnosis occurred and the type of test used were not assessed. Self-testing presents challenges to document to what extent reactive results are being confirmed and linked to care and to quantify what is their contribution to the overall number of newly diagnosed individuals. However, unless surveillance units publish data on setting and type of test used for newly diagnosed individuals, it seems as a natural step that needs to be taken in future studies on HIV self-testing.

Awareness and use of HIV self-testing among MSM recruited online in Spain, has increased when compared to studies conducted before its legal authorization but remains low. In order to reach its full potential, the use of self-tests needs to be increased by improving awareness especially among less favored MSM such as Latin-American migrants, with lower education levels, who are undertested and less open about their sexuality. Additionally, the distribution and sale of self-testing through different ways could also contribute to increase its use.

## Data Availability Statement

The original contributions presented in the study are included in the article/supplementary material, further inquiries can be directed to the corresponding author/s.

## Methysos Project Group

María del Carmen Burgos, César Pérez Romero, Lidia Herrero (Instituto de Salud Carlos III, Madrid); José Antonio San Juan Bueno (Asociación Pink Peace, Madrid); David Palma Díaz, Francisca Roman Urrestaruzo, Jesus E Ospina, Miguel Alarcón Gutiérrez (Agència de Salut Pública de Barcelona, Barcelona); Jorge del Romero, Oskar Ayerdi, Carmen Rodríguez, Sonsoles del Corral Del Campo, Natividad Jerez Zamora, Marta Ruiz Fernández, Montserrat González Polo (Centro Sanitario Sandoval, Madrid); María Jesús Barbera Gracia, Jorge-Néstor García-Pérez, Luis López Pérez, Claudia Broto Cortes, Julio Morais Martin (Unitat d'Infeccions de Transmissió Sexual Drassanes, Hospital de la Vall d'Hebron, Barcelona).

## Author Contributions

LF, PG, and M-JB conceived, designed, and supervised the study. J-MG and PG organized the recruitment of participants. J-MG, MD, JP, and LS contributed to the recruitment of subject and data collection. J-MG, JH, MD, LS, and JP performed the main analyses. J-MG, JH, PG, and M-JB wrote the manuscript. JH, LF, and M-JB were responsible for drafting and critical revisions of the manuscript. J-MG, JH, LF, MD, JP, LS, PG, M-JB, and the additional members of the Methysos Project Group made substantive contributions to the current article. All authors read and approved the final manuscript. All authors contributed to the article and approved the submitted version.

## Funding

This work received funding from the National Drugs Plan (Ministry of Health, Government of Spain, 2019I017).

## Conflict of Interest

The authors declare that the research was conducted in the absence of any commercial or financial relationships that could be construed as a potential conflict of interest.

## Publisher's Note

All claims expressed in this article are solely those of the authors and do not necessarily represent those of their affiliated organizations, or those of the publisher, the editors and the reviewers. Any product that may be evaluated in this article, or claim that may be made by its manufacturer, is not guaranteed or endorsed by the publisher.
